# Drug-Drug Interactions of Irinotecan, 5-Fluorouracil, Folinic Acid and Oxaliplatin and Its Activity in Colorectal Carcinoma Treatment

**DOI:** 10.3390/molecules25112614

**Published:** 2020-06-04

**Authors:** Marloes Zoetemelk, George M. Ramzy, Magdalena Rausch, Patrycja Nowak-Sliwinska

**Affiliations:** 1 Molecular Pharmacology Group, School of Pharmaceutical Sciences, Institute of Pharmaceutical Sciences of Western Switzerland, University of Geneva, 1 Rue Michel-Servet, 1211 Geneva 4, Switzerland; Marloes.Zoetemelk@unige.ch (M.Z.); george.ramzy@unige.ch (G.M.R.); Magdalena.Rausch@unige.ch (M.R.); 2 Translational Research Center in Oncohaematology, University of Geneva, 1 Rue Michel-Servet, 1211 Geneva 4, Switzerland

**Keywords:** colorectal carcinoma (CRC), drug-drug interaction, optimized drug combination, treatment schedule, synergy

## Abstract

The combination of folinic acid, 5-fluorouracil, oxaliplatin and/or irinotecan (FOLFOXIRI) is the standard of care for metastatic colorectal cancer (CRC). This strategy inhibits tumor growth but provokes drug resistance and serious side effects. We aimed to improve FOLFOXIRI by optimization of the dosing and the sequence of drug administration. We employed an orthogonal array composite design and linear regression analysis to obtain cell line-specific drug combinations for four CRC cell lines (DLD1, SW620, HCT116, LS174T). Our results confirmed the synergy between folinic acid and 5-fluorouracil and additivity, or even antagonism, between the other drugs of the combination. The drug combination administered at clinical doses resulted in significantly higher antagonistic interactions compared to the low-dose optimized drug combination (ODC). We found that the concomitant administration of the optimized drug combination (ODC) was comparatively active to sequential administration. However, the administration of oxaliplatin or the active metabolite of irinotecan seemed to sensitize the cells to the combination of folinic acid and 5-fluorouracil. ODCs were similarly active in non-cancerous cells as compared to the clinically used doses, indicating a lack of reduction of side effects. Interestingly, ODCs were inactive in CRC cells chronically pretreated with FOLFOXIRI, suggesting the occurrence of resistance. We were unable to improve FOLFOXIRI in terms of efficacy or specificity. Improvement of CRC treatment should come from the optimization of targeted drugs and immunotherapy strategies.

## 1. Introduction

Colorectal cancer (CRC) is the third most commonly diagnosed cancer with an incidence of 1.8 million cases in 2018, which is expected to reach approximately 2.2 million worldwide by 2030 [1]. With a mortality rate of 700,000 patients per year worldwide, it also has the fourth most cancer-related deaths [2,3]. The 5-year survival rate is 21% for all races in late-stage CRC, compared to 65% in early-stage diagnosis [4]. The choice of current clinical management of CRC depends on the stage of the disease, the molecular analysis of the tumor, i.e., microsatellite instability, KRAS and BRAF mutations [5,6], as well as the health status of the patient [7]. Patients with local early-stage tumors undergo surgical resection. Late-stage or metastatic lesions very often cannot be removed by surgical resection and patients generally receive chemotherapy, applied as a combinatory treatment [8].

**Figure 1:**
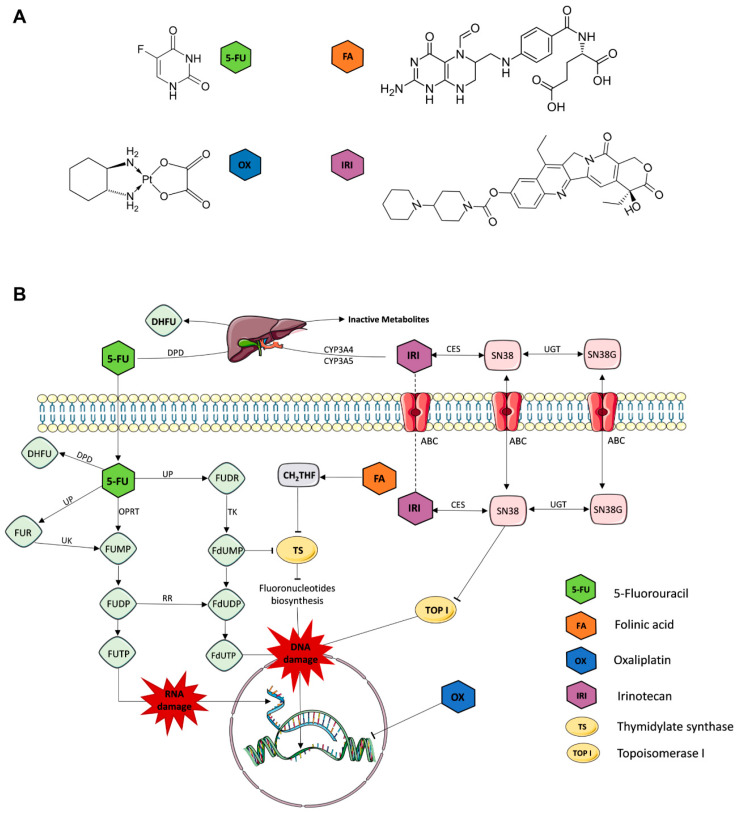
Chemotherapeutic drugs for the treatment of colorectal cancer (CRC) and their mechanisms of action. (**A**) Chemical structures of folinic acid (FA), 5-fluorouracil (5-FU), irinotecan (IRI) and oxaliplatin (OX). (**B**) Graphical representation of the mechanisms of action of each drug. 5-FU is extensively processed into FUMP (fluorouridine monophosphate) directly by the enzyme OPRT (orotate phosphoribosyl transferase) or indirectly via UP and UK (phosphorylase and uridine kinase) processing of FUR (5-fluorouridine). FUMP is further converted into FUTP (fluorouridine triphosphate) and FdUTP (5-fluorodeoxyuridine triphosphate), two active metabolites that cause misincorporation of fluoronucleotides, promoting RNA and DNA damage. Another active metabolite is created through the processing of 5-FU into FdUMP (fluoro-deoxy uridylate) via FUDR (fluorodeoxyuridine) by UP and TK (tyrosine kinase). FdUMP strongly competes with dUMP (deoxyuridine monophosphate) for TS (thymidylate synthase), thereby inhibiting fluoronucleotide biosynthesis needed for DNA synthesis and replication. Alternatively, 5-FU is reduced to substantially less toxic DHFU (5,6-dihydro-fluorouracil) by DPD (dihydro-pyrimidine dehydrogenase). FA is converted into CH_2_THF, which stabilizes TS, similar to FdUMP, and prevents natural substrate dUMP from binding to TS. OX directly forms platinum adducts with DNA, thereby inhibiting DNA replication. IRI is metabolized by liver enzymes (mainly CYP3A4 and CYP3A5) into SN-38 and SN-38G by CES and UGT (carboxylesterases and uridine diphosphate glucuronosyltransferase). IRI and its more active metabolite SN bind to topoisomerase I and DNA complexes to prevent DNA replication and induce DNA damage.

For decades, the chemotherapeutic drug 5-fluorouracil (5-FU) has been the backbone of therapy for CRC and the standard first-line treatment for metastatic CRC (mCRC) [9]. 5-FU is extensively converted intracellularly into multiple active metabolites, such as 5-fluorouridine 5′-triphosphate (FUTP) and 5-fluoro-2′-deoxyuridine 5′-triphosphate (FdUTP), which disrupt the synthesis of RNA via the miss-incorporation of fluoronucleotides in both RNA and DNA. Simultaneously, the metabolite 5-fluoro-2′-deoxyuridine 5′-monophosphate (FdUMP) inhibits the enzyme thymidylate synthase, which orchestrates the main reactions providing thymidylate, necessary for DNA replication and repair, see Figure 1B [10]. Since the introduction of 5-FU in 1957, its combination with several chemotherapeutic agents has been widely used in clinical practice [9]. Folinic acid (FA, leucovorin) derivatives potentiate the cytotoxic effects of 5-FU through the inhibition of fluoronucleotide biosynthesis by competing with the natural substrate of thymidylate synthase (Figure 1B). Simultaneous administration of 5-FU and FA has been shown to improve response rates in CRC when compared to individually administered 5-FU bolus injections, in addition to increasing patients’ median and overall survival [11,12]. Oxaliplatin (OX), a third-generation platinum-based agent forming platinum-DNA adducts (Figure 1B), triggers the immobilization of the mitotic cell cycle, inducing apoptosis [13]. Finally, irinotecan (IRI), also known as CPT-11, is a topoisomerase I inhibitor. While CPT-11 only marginally inhibiting DNA synthesis by itself, it is processed by liver enzymes into the active metabolite SN-38 (SN). SN-38 binds to topoisomerase I and DNA complexes, resulting in the formation of stable ternary structures and playing an essential role in CPT-11-mediated anti-tumor activity (Figure 1B). These structures promote DNA damage by inducing chromatid breaks directly or indirectly by colliding with moving replication forks during S-phase, ultimately leading to apoptosis [14,15], see Figure 1 and Table 1.

Mixtures of FA/5-FU with either OX or IRI (called FOLFOX or FOLFIRI) or a mixture of all four (called FOLFOXIRI) were introduced as the standard of care, of which FOLFOXIRI is the most commonly used chemotherapy combination applied sequentially [16,17,18,19]. Several clinical trials were reported to determine the benefits of FOLFOXIRI for patients with advanced or metastatic CRC [19,20,21,22,23,24,25], but others show no benefit over FOLFIRI and observed significantly more side effects such as high incidence of alopecia, neutropenia, nausea/vomiting, diarrhea, and considerable rates of febrile neutropenia [20,24,26,27]. The treatment efficacy remains low for the majority of patients because of treatment intolerance or resistance, which in most cases results in a reduced life expectancy. Until now, chemotherapy is reported to be the most effective, yet aggressive and non-personalized, treatment option for inoperable CRC. Efforts have been made to improve chemotherapy effectiveness and tolerance by including treatment pauses, or by applying the treatment in a determined schedule. Nevertheless, the overall outcome is the risk of significant toxicity and a low overall survival rate [28].

In this study, we analyzed the drug-drug interactions within the FA, 5-FU, OX, and SN (the active metabolite of IRI) combination (FA/5-FU/OX/SN) in human colorectal carcinoma cell lines. We chose the combination of FA/5-FU/OX/SN due to higher overall and progression-free survival in patients with mCRC, compared to standard chemotherapy regimens [20,29]. We used predefined matrices called ‘orthogonal array composite designs’ for experimental testing of multidrug combinations and a second-order linear regression model for data analysis [30]. This robust and validated approach was previously used to identify multidrug combinations of targeted compounds for the treatment of renal cell carcinoma or colorectal carcinoma [30,31,32]. Through this approach, we were able to establish low-dose optimized drug combinations (ODCs) of FA/5-FU/OX/SN and their activity was further compared to the drug combination applied at higher, clinically used doses (CUD). In contrast to the CUD, the ODCs demonstrated synergistic and additive drug interactions. The sequential administration of these ODCs did not enhance treatment efficacy. Moreover, CRC cells chronically pre-treated with ODC showed significantly reduced sensitivity to FA/5-FU/OX/SN treatments, representing one of the major issues, i.e., acquired treatment resistance, in clinical circumstances.

## 2. Results

### 2.1. Optimization of Cell Line-Specific Low-Dose Drug Combinations

**Figure 2:**
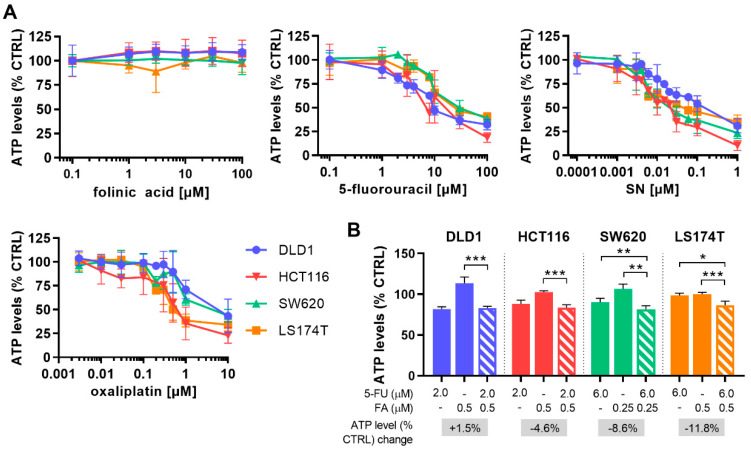
Drug dose-response curves for folinic acid, 5-fluorouracil, SN and oxaliplatin and the optimization of combining folinic acid and 5-fluorouracil. (**A**) Drug response curves of folinic acid (FA), 5-fluorouracil (5-FU), SN-38 (SN) and oxaliplatin (OX) in all CRC cell lines presented on a logarithmic scale. Cells were treated for 72 h. (**B**). Drug dose optimized for FA and 5-FU towards the combined activity of 20% on cell metabolic activity (ATP) inhibition after 72 h treatment. The increase in cell metabolic activity inhibition of the FA/5-FU (FF) combination compared to 5-FU alone is indicated in the grey box. Error bars represent the SD for N = 2–6 experiments (**A**) or N = 3 experiments (**B**) and significances of * *p* < 0.05, ** *p* < 0.01 and *** *p* < 0.001 represent the comparison of single drugs with FA and 5-FU combined as determined by a two-way ANOVA with post-hoc Dunnett’s multiple comparisons test.

In the first step, dose-response curves for each drug were established in four human CRC cell lines (DLD1, HCT116, SW620, and LS174T). The cell lines differed in biological characteristics such as genetic stability and mutation status (Table 1). Drug activities were compared by measuring the cell metabolic activity, presented as % of control (sham-treated, CTRL), which indirectly corresponds to cell viability. All cell lines marginally varied in their sensitivity to all chemotherapeutic drugs and 5-FU, SN, and OX diminished ATP levels in a dose-dependent manner, whereas the vitamin FA was inactive in the range of concentrations tested (Figure 2A).

The dose-response curves generated for each drug in each cell line, (Figure 2A) were used to select for doses corresponding to 20% and 10% inhibition of the cell metabolic activity (ED_20_ and ED_10_). These are doses that allow the determination of drug-drug interactions in the course of the low-dose drug combination optimization. As FA and 5-FU are, without exception, clinically administered simultaneously, FA and 5-FU were combined as a monotherapy (FF). FA was previously reported to stabilize the 5-FU target enzyme thymidylate synthase, thereby enhancing the activity of 5-FU [11,12]. In our study, FA induced an increase in the activity of 5-FU, enhancing the inhibition significantly with 8.6% for SW620 and 11.8% for LS174T, see Figure 2B. This was not the case in DLD-1 or HCT116 cells.

We calculated the combinatory index (CI) to describe the potential drug interactions for the FA/5-FU combination, shown as synergistic (CI < 1), additive (CI = 1), or antagonistic (CI > 1) activities. Synergistic interaction of FF was observed in SW620 and LS174T, additivity in HCT116, and antagonistic interaction in DLD1 cells (Table 2, Supplementary Figure S1 and Supplementary Table S1).

**Figure 3:**
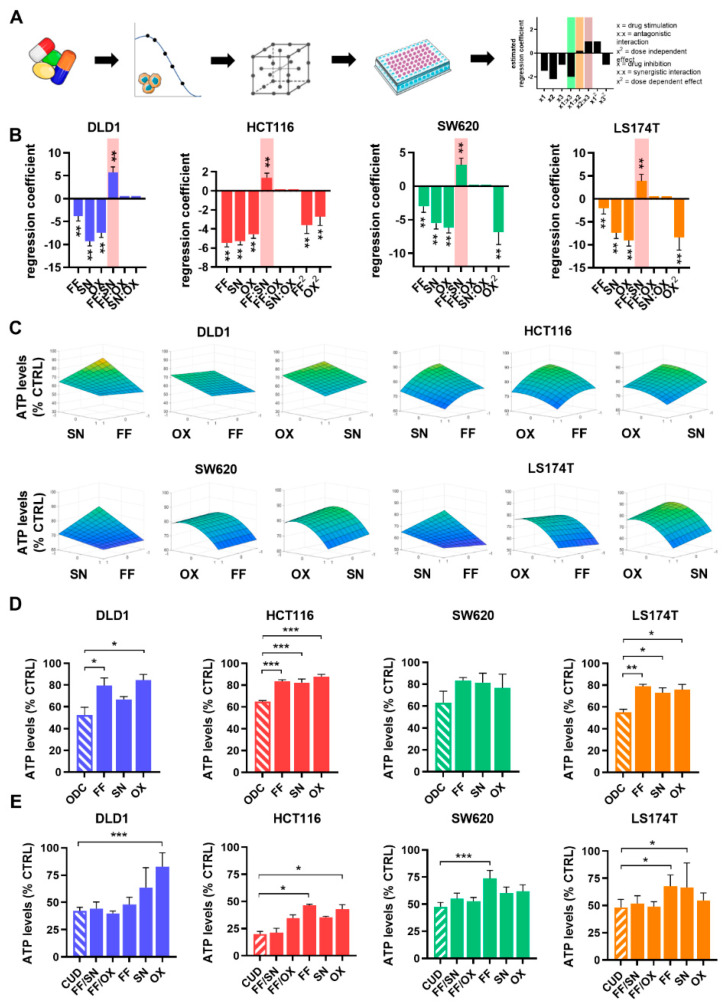
Optimization of low dose optimized drug combinations (ODCs) and validation of the clinically used doses (CUD) combination. (**A**) Schematic representation of the optimization process. Starting with the drugs, namely folinic acid and 5-fluorouracil combined (FF), SN-38 (SN) and oxaliplatin (OX), in the first step, drug dose-response curves were generated and low doses inhibiting with approximately 20% cell metabolic activity (ATP) were selected as drug dose input for the next step (FF, SN, OX). Drug combinations were screened according to a predefined orthogonal array composite design (OACD) matrix in cells seeded in 96-well plates and drug combination activity on cell metabolic activity was modeled based on step-wise second-order linear regression analysis. The models present regression coefficients identifying the contribution of single-drug activity of each drug and drug-drug interactions contributing to the overall activity of the combination. (**B**) Stepwise second-order linear regression model depicting the regression coefficients of the drugs composing the ODC at low doses for each of the cell lines, screened concomitantly (Schedule 1). (**C**) Response surface contour plots between FF, SN and OX, fitted with the regression coefficients generated. The *y*-axis represents drug efficacy (ATP levels, % CTRL), the *x*-axis represents the dose range (1, high dose, ED_20_; 0, low dose, ED_10_; −1, no drug) for each drug. (**D**) Efficacy of drug combinations and corresponding monotherapies of the cell-specific ODCs and each monotherapy at an equal concentration as in the corresponding ODC. (**E**) The drug combinations at clinically used doses (CUD). Error bars represent the SD for N = 2–3 experiments and * *p* < 0.05, ** *p* < 0.01 and *** *p* < 0.001 represents the significance of estimated regression coefficients (**B**) or the comparison with the full drug combination of N = 2–4 experiments (**D**,**E**) by a one-way (**B**,**D**) or two-way ANOVA (**E**) with post-hoc Dunnett’s multiple comparisons test.

To determine the optimal drug-dose and drug-drug interaction between FA, 5-FU, OX, and SN, we employed predefined matrices called orthogonal array composite designs (OACD, see Materials and Methods). Screening of the drug combinations was performed according to the OACD matrix and the resulting inhibition of cell metabolic activity was used to model drug combination activity using step-wise second-order linear regression analysis (Figure 3A). This process generated regression coefficients describing the relationship between a predictor variable and the response. The regression coefficients allow the estimation of the contribution of each drug separately (single-drug first-order term), as well as part of a drug pair (drug-drug interaction term) to the overall activity of the drug combination. The single-drug quadratic term, describing the dose-dependency of the drug contribution, was also considered. Overall, negative values of the regression coefficients indicate drug inhibition or synergism (bar highlighted in green) and that an increased dose of that drug potentiates cell metabolic activity inhibition. Inverted, positive regression coefficients indicate drug stimulation or antagonism (bar highlighted in red) or additivity (bar close to 0, highlighted in orange), and stable inhibition of a drug across the dose range tested, see Figure 3A.

In each CRC cell line, antagonistic drug-drug interactions were observed between FF and SN (Figure 3B, bar highlighted in red) and none of the evaluated drug interactions were synergistic. In accordance, the CI indicated antagonism for the overall combination of all cell lines (Table 3, Supplementary Figure S2 and Supplementary Table S2). The strongest single drug contributions were derived from SN and OX (Figure 3B). Furthermore, OX had the strongest dose-dependent effect.

Drug combination efficacy is related to the applied drug doses by quadratic response surfaces [38]. Visual representation of response surfaces representing the 3-dimensional relation between efficacy (*z*-axis) and the dose range of two drugs’ (*x* and *y* axes) were built based on the regression coefficients (Figure 3C). The fact that those response surfaces are smooth confirms that the low-dose ODCs are the most optimal across all possibilities [39,40]. These results confirmed the overall efficacy (35–48%) of the drug combinations on the CRC cells with the drugs interacting antagonistically and/or additively, and identified the optimized drug combinations (ODCs) at low doses (Figure 3D).

As a positive control, we used three-drug (FA/5-FU/SN or FA/5-FU/OX) or four-drug (FA/5-FU/OX/SN) combinations applied at clinically used doses (CUD), converted to in vitro units (see Section 4). The ODCs were administered 24 h after cell seeding and kept concomitantly for 72 h (Figure 3D, Schedule 1). The doses of the ODCs were mostly much lower compared to the CUD, especially for 5-FU (5-fold in DLD1 and HCT116 cells) or SN (33-fold in HCT116 cells), see Table 3. All ODCs presented a more potent activity than the corresponding monotherapies. In contrast to the CUD, the activity of the ODC is not promoted by one drug only, but by the synergistic or additive interplay of the combined drugs at optimized doses. The four-drug combination FA/5-FU/OX/SN inhibited the ATP levels up to 80%, (HCT116 cells, Figure 3E, administrated concomitantly, Schedule 1). Of note, the three-drug combinations FA/5-FU/SN and FA/5-FU/OX, administered at CUD, were similarly active to the four-drug combination FA/5-FU/OX/SN. The activity of monotherapies at corresponding doses was mild to strong (e.g., 60% for SN and OX in HCT116 cells), Figure 3E.

**Figure 4:**
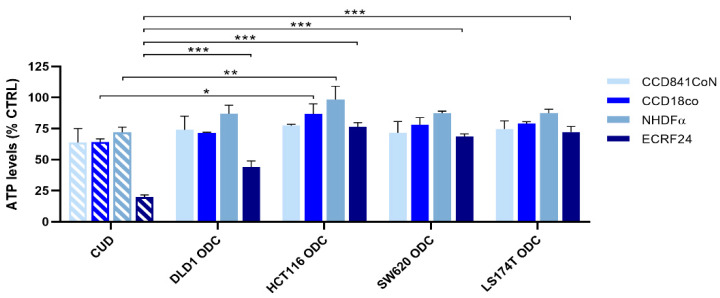
Validation of the drug combinations in non-cancerous cell lines. Validation of the clinically used dose (CUD) combination and each of the cell-specific ODCs at low doses in various non-cancerous cell lines presented with drug combination efficacy on cell metabolic activity. Error bars represent the SD for N = 2–3 experiments and significances of * *p* < 0.05, ** *p* < 0.01 and *** *p* < 0.001 represent the comparison with the CUD combination for all cell lines by a one-way ANOVA with post-hoc Dunnett’s multiple comparisons test.

In order to evaluate the toxicity of the drug combinations, each cell line-specific ODC was tested in a panel of non-cancerous cell lines, i.e., human colon epithelial cells (CCD841coN), human colon fibroblasts (CCD18co) and human immortalized macrovascular endothelial cells (ECRF24). The ODCs showed 2–25% of activity towards normal fibroblasts and epithelial cells, Figure 4. This activity was reduced significantly in HCT116 cells as compared to the CUD combination. Importantly, all ODCs presented lower activity on endothelial cells than the CUD combination.

### 2.2. The Search for Optimal Drug Administration Sequence

**Figure 5:**
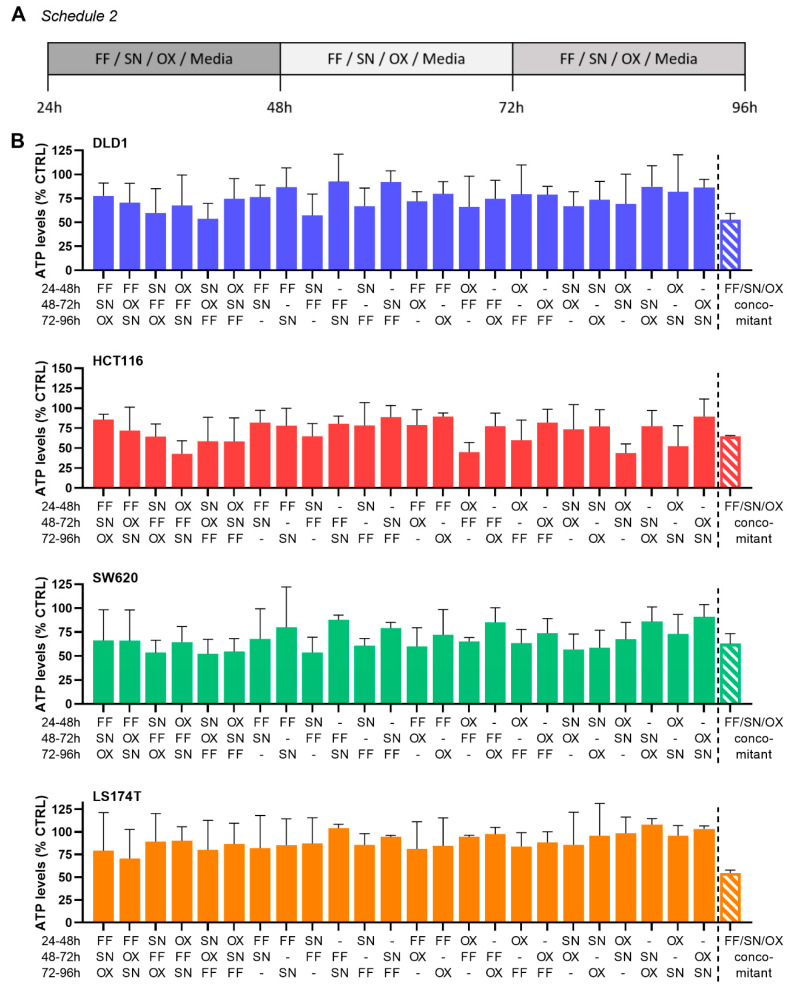
Sequential drug administration of the ODCs. (**A**) Schematic representation of the sequential drug administration of folinic acid and 5-fluorouracil combined (FF), SN-38 (SN), oxaliplatin (OX) and medium in 24 h intervals (Schedule 2), and (**B**) the drug combination efficacy on cell metabolic activity for each of the cell lines. Error bars represent the SD for N = 3 experiments. No statistically significant difference was observed between the conditions tested.

To determine the optimal order of sequential administration of each drug resulting in the highest activity of the ODC, CRC cells were exposed to drugs sequentially, starting 24 h after cell seeding (Figure 5A, Schedule 2). The cells were incubated with either one of the drugs (FF, SN and/or OX) or medium. After 24 h, the alternating treatment conditions were replaced with either another drug or medium, depending on the schedule. Administration of SN as the first drug seemed to sensitize the cells to the drugs subsequently administered and induced the highest inhibition in DLD1 (43%) and SW620 (47%) cells. The most potent effect (49%) in HCT116 cells was observed when OX was administered before other drugs. For LS174T cells, the most effective sequence of drug administration was the one initiated with FF (25%), Figure 5B. The drug administration according to this schedule did not reveal better efficacy as compared to concomitant drug administration (Schedule 1).

### 2.3. Integration of Cell Line-Specific ODCs in Clinically Used Drug Administration Schedules

**Figure 6:**
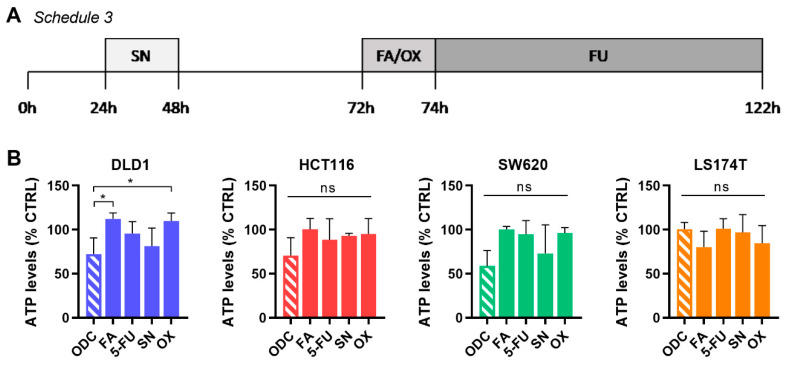
Validation of the drug combinations in a clinically used drug administration schedule. (**A**) Schematic representation of an intermittent drug administration schedule extrapolated from a clinical setting (Schedule 3). CRC cells were seeded and kept without treatment for 24 h. Afterward, the culture medium was removed to apply SN for 24 h, followed by the removal of used cell culture medium and addition of fresh medium for another 24 h. Starting at 72 h post-experiment initiation, the cell culture medium was replaced by a combination of FA/OX, kept for 2 h, and then replaced by 5-FU for another 48 h. The entire procedure lasted for 122 h. (**B**) Drug combination efficacy on cell metabolic activity in each of the cell lines. Error bars represent the SD for N = 3 experiments and significances of * *p* < 0.05 represent the comparison with the full combination for all cell lines by a one-way ANOVA with post-hoc Dunnett’s multiple comparisons test.

In the clinical setting, varying administration regimens for each drug in the combination are applied, mostly due to different drug pharmacokinetic profiles. Commonly, OX, FA and IRI are administrated intravenously for 1.5–2 h followed by a bolus injection of 5-FU with continuous infusion of 5-FU for another 48 h. The treatment procedure lasts approx. 52 h, depending on the initial sequence, and is repeated in cycles once weekly for up to 8 weeks [6,41]. This treatment schedule was previously extrapolated to in vitro settings [42], Schedule 3, Figure 6A.

In our experiments, CRC cells were seeded and kept without treatment for 24 h. Afterward, the culture medium was removed to apply SN for 24 h, followed by the removal of used cell culture medium and addition of fresh medium for another 24 h. Starting at 72 h post-experiment initiation, the cell culture medium was replaced by a combination of FA and OX, kept for 2 h, and then replaced by 5-FU for another 48 h. The entire procedure lasted for 122 h. Interestingly, while only applied for 24 h, SN (DLD1 and SW620) was still more active than 5-FU for 48 h at the end of the schedule. In general, the activity of all ODCs was inferior to the results obtained using Schedule 1 or 2 (Figure 6B).

### 2.4. Chronically Pretreated Colorectal Carcinoma Cells Lose Sensitivity to FA/5-FU/OX/SN Combinations

**Figure 7:**
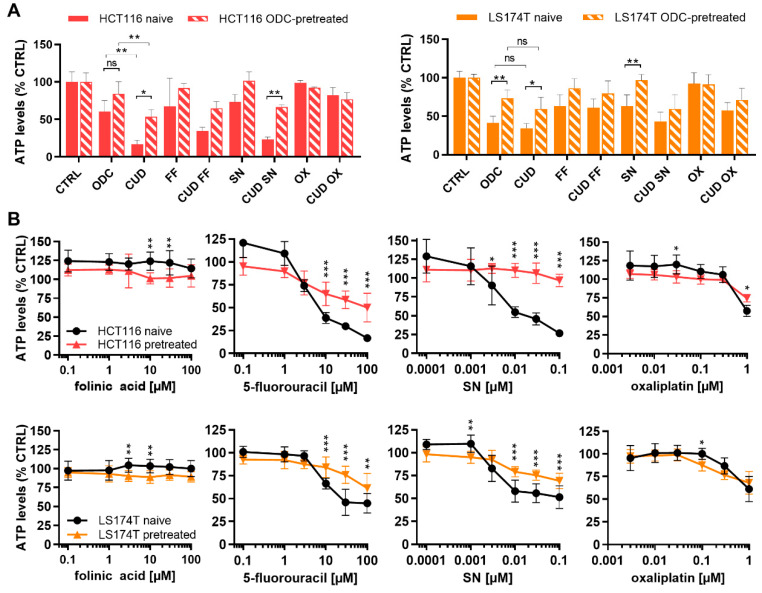
Drug combination activity in chronically ODC-pretreated cells. (**A**) Drug combination efficacy of the ODC and CUD on cell metabolic activity in HCT116 and LS174T naïve and FA/5-FU/SN/OX pretreated cells (HCT116-pretreated and LS174T-pretreated). (**B**) Drug dose-response curves of folinic acid (FA), 5-fluorouracil (5-FU), SN and oxaliplatin (OX) in HCT116 and LS174T naïve and FA/5-FU/SN/OX pretreated cells. Drug concentrations are presented on a logarithmic scale. Error bars represent the SD and significances of * *p* < 0.05, ** *p* < 0.01 and *** *p* < 0.001 represent the comparison between naïve and pretreated cells (unpaired t-test) or the comparison between the ODC and CUD (two-way ANOVA with post-hoc Dunnett’s or Sidak’s multiple comparisons test) including (**A**) N = 3 or (**B**) N = 3–4, respectively.

Patients receiving chemotherapeutic drug combinations lose sensitivity to the treatment with time. To mimic this situation, LS174T and HCT116 cells were chronically treated with the cell-specific ODCs for >17 weeks. The efficacy of ODCs and CUD combinations was evaluated in these chronically pretreated cells. The ODC and CUD combinations could not maintain efficacy in the ODCs-pretreated cells with a respective increase in ATP levels from 60.2% to 83.9% and 16.9% to 53.5% with *p* < 0.05 for HCT116 and from 41.4% to 73.5% with *p* < 0.01 and 34.5% to 59.4% with *p* < 0.01 in LS174T cells (Figure 7A). Notably, the biggest loss in sensitivity was observed for the high dose CUD combination in the HCT116-pretreated cells. The pre-treated cells were also less sensitive to the corresponding FF and SN monotherapies, but not OX (Figure 7A). Indeed, drug dose-response curves for each of the drugs show significant loss of sensitivity of 5-FU and SN at higher doses in both HCT116-pretreated and LS174T-pretreated cells (Figure 7B), indicating resistance mechanisms appearing in both cell lines.

## 3. Discussion

Multidrug combinations used clinically for the treatment of CRC contain folinic acid (FA), 5-fluorouracil (5-FU), oxaliplatin (OX) and/or irinotecan (IRI) and show promising anti-tumor effects, but simultaneously induce drug resistance and serious side effects [43]. The main goal of this study was to identify effective cell line-specific low-dose FA/5-FU/OX/SN combinations that outperform the clinically used drug combination. We further investigated if alternative administration schedules could enhance ODC efficacy and if pre-treatment affects sensitivity to continued FA/5-FU/OX/SN treatments.

Using our validated multidrug combination optimization method together with data modeling we identified alternative optimized drug combinations (ODCs) with low FA/5-FU/OX/SN doses. When administered simultaneously, the ODCs gave similar effects to the same drug combination applied at much higher, clinically used doses (CUD), especially in DLD1 and LS174T cells. Moreover, the ODCs were optimized for each cell line, therefore fitting each CRC subtype better. Interestingly, the activity of ODCs on human endothelial cells was significantly less potent than the CUD drug combination. This important observation suggests that in in vivo conditions, drug delivery of an ODC could be facilitated, as the vasculature might not be impaired considerably. Moreover, we observed that ODCs were similarly active on non-cancerous epithelial cells to the CUD combination (Figure 4).

In all of the FA/5-FU/OX/SN combinations used in the clinics, FA and 5-FU (FF) are invariably administered together. Indeed, we also found that when administered simultaneously, the inhibition of the cell metabolic activity increased in three out of the four cell lines (Figure 2). The analysis of drug-drug interactions between FF, SN, and OX at the tested doses, did not show any synergism as demonstrated using linear regression analysis or the combination index (CI), see Table 3 and Figure 3. Without exception, FF and SN interactions were antagonistic or additive in each cell line. This is in agreement with the earlier findings of Fischel et al., who reported additive activity of FF, SN, and OX in SW620 cells [42], although the doses used within the mentioned study corresponded to the IC_50_ and were higher (3- to 20-fold) than doses optimized in the frame of this study. Generally, all cell lines seemed to be similarly sensitive to each of the drugs except for HCT116 cells, which were most sensitive to OX at doses ≤0.2 µM. HCT116 cells were also mostly affected by the drug combination administered at clinically used doses, CUD (Figure 3E). Comparing the characteristics of the four cell-lines (Table 1) to drug sensitivity (Figure 1 and Figure 7) indicates that there is no relation between specific mutations and drug sensitivity. However, as HCT116 resembles an early-stage tumor type harboring the lowest number of documented mutations, the pronounced drug sensitivity of HCT116 cells could be related to this.

Examination of the effects of the FA and 5-FU combination revealed the only synergistic interaction observed in this study. Applying drug administration schedules with drugs administered sequentially for 24 h (Schedule 2) or 2 h (schedule 3) aimed to improve the sensitization of cells to the treatment regimens did not improve the treatment efficacy significantly over concomitant administration (Schedule 1). It seems, however, that administration of SN or OX before other drugs could sensitize the cells to other drugs marginally in a cell line-dependent manner (Figure 5), enhancing the overall efficacy. This observation is in line with findings of Mans et al., who reported that pre-treatment of SW620 cells with the IC_20_ of irinotecan for 24 h improved the growth-inhibitory effects of 5-FU by 2-fold via a synergistic interaction [44]. Also, Guichard et al. reported improved anti-tumor efficacy by administrating irinotecan before 5-FU, but also noted increased toxicity [45]. Moreover, other studies have also suggested an inverse relationship, i.e., pre-treatment with 5-FU mediated downregulation of P-glycoprotein and breast cancer resistance protein, correlated with enhanced SN absorption and efficacy [46]. This is supported by clinical observations but was simultaneously correlated with reduced tolerability [47].

To mimic a potential activity of ODC and CUD in patients already pretreated with chemotherapy, we have chronically treated HCT116 or LS174T cells with their corresponding ODC for over 17 weeks. Insensitivity to the CUD and ODC was shown in both HCT116 and LS174T-pretreated cells (Figure 7A). Moreover, the chronically pretreated HCT116 cells showed considerably reduced sensitivity to high doses of 5-FU and SN compared to treatment-naïve cells. Previously, Haug et al. have reported that HCT116 cells develop treatment resistance to 5-FU, without leading to cross-resistance with irinotecan [45], indicating the involvement of different mechanisms of resistance. Those include induction of anti-apoptotic and survival signaling [48,49,50], alterations in topoisomerase I levels or complex formation [51], or high levels of thymidylate synthase (TS) [52]. The latter was also reported in LS174T cells and is in line with our findings of reduced sensitivity to 5-FU due to pretreatment. Moreover, while a variety of resistance mechanisms have been reported for OX [13], pretreatment with the cell-specific ODCs did not induce loss of sensitivity to OX. Resistance to FA has also been reported; the target of FA, TS, is a key enzyme for DNA biosynthesis [53]. Resistance to FA-mediated potentiation of 5-FU has been reported to be associated with decreased stability of TS [54,55], partially explaining the intrinsic differences in potentiation and synergy of FA observed between the cell lines (Figure 2B). However, pretreatment-mediated loss of sensitivity to 5-FU was not further dependent on FA (Figure 7B).

Poor tolerability of FOLFOXIRI occurs in a significant proportion of patients [20,24]. Multiple clinical studies for colorectal and pancreatic cancer have been conducted to analyze the tolerability of FOLFOXIRI.Particularly for pancreatic cancer, FOLFOXIRI in combination with gemcitabine, e.g., PORIDGE-24 trial, is now applied [56]. A phase II clinical trial initiated in 2019 (NCT03977233) was designed to measure the impact of FOLFOXIRI on genetically different pancreatic cancer subtypes, following a personalized chemotherapeutic approach [57]. Moreover, various alternative approaches for the treatment of pancreatic cancer are being explored to establish new treatment options and therapeutics i.e., gentamicin in combination with immunotherapy (a monoclonal antibody against CD40) [58] or metabolic inhibitor CPI-613 (Devimistad) [59]. Similar approaches could be investigated to improve CRC treatment.

FOLFOXIRI remains the most applied and important first-line treatment of mCRC, further guiding treatment decisions [60]. Despite the existence of greater toxicity, it remains manageable [29]. The treatment with chemotherapy combination turns non-resectable aggressive tumors into resectable lesions and changes the molecular pattern of the tumor [9]. At this stage, targeted treatments become effective [60]. The balance between treatment success and patient well-being remains indefinable for chemotherapy combinations with or without the addition of targeted treatments. The replacement of FOLFOXIRI with other first-line treatment strategies is currently under development for the treatment of (metastatic) CRC. Treatment for stage IV CRC is proposed using various strategies such as (i) the combination of capecitabine and oxaliplatin [61], (ii) capecitabine, with or without a targeted drug (NCT00642603) [62,63], (iii) cetuximab (epidermal growth factor receptor inhibitor) [64] and (iv) regorafenib (multi-target kinase inhibitor) [65] or bevacizumab (Avastin^®^) [26,27,66].

The introduction of combinations of non-chemotherapeutic drugs, as well as immunotherapeutic regimens [67], have raised interest and might be beneficial for the treatment of CRC. Immunotherapy with antibodies directed against programmed death (PD-1) or programmed death-ligand 1, PD-L1 (nivolumab and pembrolizumab, respectively) have been approved by the FDA in 2017 for the treatment of immunogenic deficient mismatch repair or high levels of microsatellite instability tumors, including CRC [68,69], and should continue to be investigated. To date, the management of mCRC with chemotherapy as the backbone of treatment has been considered palliative for many years, with little expectations of cure. This paves the way to potential new therapies to replace or combine with the current standard of care. A combination of FOLFOXIRI with immunotherapy may revolutionize standard clinical care [70,71,72]. The presence of immune cells in the tumor microenvironment of mCRC after first-line chemotherapy treatment can be used to predict potential combinations with immunotherapy [72,73,74]. FOLFOX or FOLFOXIRI treatment triggers immune cell infiltration [74], increases the expression of immune-modulatory receptors and can prime and enhance for some time the immune cell activity at the tumor site [72,74]. Interestingly, it has been demonstrated that FOLFOX treatment upregulates the expression of PD-L1 on cancer cells, inducing immune resistance. In this context, the combination of FOLFOX with anti-PD-1 therapy showed enhanced efficacy overcoming the prior induced resistance [71]. At the moment, combinations of FOLFOXIRI and immunotherapy are being tested in clinical trials in advanced pancreatic cancer [75]. The ultimate goal of these initiatives is to improve clinical outcomes while reducing side effects and improving the patient’s quality of life.

We believe that the study supports therapeutic implications. Firstly, the study provides further evidence that the original composition of chemotherapy combination was not necessarily based on a rational design in terms of drug-drug interactions, thus reducing the overall benefit of administrating the drugs together. We further underline this point by loss of sensitivity after long-term pre-treatment of cells with this drug combination. Secondly, we aimed to improve the drug combination activity and selectivity by reducing the drug doses or adjusting the drug administration sequence, but with limited success. Therefore, we emphasize the importance of a balance in the treatment decision that should be against less toxic and more efficient treatment options available. Such an individualized approach was shown to be an effective strategy for pancreatic cancer treatment.

Finally, we argue that FA/5-FU/OX/SN or a variation of it could be very useful in combination with alternative or innovative treatment strategies. For example, FOLFOXIRI treatment resulting in significant amounts of cell death in early treatments could provide a source of tumor antigens. Simultaneously inducing anti-tumor immune responses through immune therapy could result in an improved overall outcome.

## 4. Materials and Methods

### 4.1. Cells and Cell Culture Conditions

Human CRC and non-cancerous colon CCD841CoN and CCD18co cell lines were obtained from ATCC or Public Health England with a corresponding authentication certificate. Human immortalized endothelial cells ECRF24 [76] were generously donated by Prof. AW Griffioen (Angiogenesis Laboratory, UMC Amsterdam). Normal human dermal fibroblast adult (NHDFα) cells were purchased from Vitaris (Baar, Zug, Switzerland). Cells were cultivated at 37 °C in a humidified atmosphere with 5% CO_2_ in corresponding culture media supplemented with 10% fetal bovine serum (S1810-500, Biowest, Nuaillé, France) and 1% penicillin/streptomycin (4-01F00-H, Bioconcept, Basel, Switzerland). HCT116, LS174T, and SW620 cells were cultured in DMEM Glutamax medium (31966-021, Gibco, Gaithersburg, MD, USA), DLD1 in RPMI-1640 Glutamax medium (1870-010, Gibco), ECRF24 in DMEM/RPMI 1:1 on a 0.2% gelatin-coated surface (G1393, Sigma-Aldrich, Buchs, Switzerland) and CCD841 and CCD18co in EMEM medium (M2279, Sigma-Aldrich) supplemented with 2 mM L-Glutamin (25030024, Gibco). Cells were tested for mycoplasma contamination frequently and authenticated by Microsynth AG (Balgach, St. Gallen, Switzerland). Cell line identity was confirmed using STR systems from Promega (Zurich, Switzerland) and database comparison.

To generate FF/SN/OX pretreated cells, HCT116 and LS174T cells were chronically exposed once weekly to their corresponding ODC for >17 weeks (concomitant administration, Schedule 1). Treatment was maintained for 72 h, then replaced with fresh medium till the next time point. Cells were kept in a T75 culture flask at higher density than the density used for naïve cells as they grew slower. The decrease in cell sensitivity to chemotherapy was evaluated every two weeks using the metabolic activity assay and compared to the parental, treatment-naïve cells.

### 4.2. Drugs, Treatments and Metabolic Activity Assay

10 mg/mL 5-fluorouracil (F6627, Sigma-Aldrich) and 1 mg/mL SN38 (29112, MedChem Express, Monmouth Junction, NJ, USA) were dissolved in sterile DMSO (Sigma-Aldrich), and 20 mg/mL folinic acid (F787, Sigma-Aldrich) and 5 mg/mL oxaliplatin (O9512, Sigma-Aldrich) in UltraPure distilled sterile water. Aliquots were stored at −80 °C and thawed before each experiment for one-time use. A maximal concentration of 0.08% DMSO in cell culture media was used as control (sham). Cells were seeded in 96-well plates at different densities (2500 cells/well for HCT116 and DLD1, 3500 cells/well for LS174T and 5000 cells/well for SW620). 24 h post-seeding single drugs or pre-mixed drug combinations were incubated for 72 h. Cell metabolic activity (ATP) was measured using the bioluminescent-based CellTiter-Glo^®^ assay (G7572, Promega) according to the manufacturer’s instructions. The intensity of the luminescence signal was detected via the BioTek Cytation 3 imaging reader with corresponding Gen5 Image software version 3.04.

### 4.3. Orthogonal Array Composite Design and Linear Regression Model

The orthogonal array composite design (OACD) is a resolution IV matrix [77] that can be used to estimate each variable’s main effect. The matrix [30,40] consists of two parts: (i) a two-level fractional factorial design to identify linear effects of single drugs and drug-drug interactions at one dose level, and (ii) a three-level orthogonal array design with both linear and second-order dose levels to cross-validate between the two parts of the matrix and identify linear response surfaces. Selectively, drug combinations were tested according to the matrix and linear regression analysis was performed to identify the drug interactions within the drug combination [30,40,78]. In practice, dose-response curves were established in the first step using a cell metabolic activity (ATP) assay for each drug in each cell line to guide the dosage to be tested in the combination corresponding to ED_20_ values and half of this dose. Next, three-drug combination in vitro screening was performed with folinic acid and 5-fluorouracil combined, oxaliplatin and SN-38 according to the OACD matrix. Finally, the metabolic activity (ATP, % CTRL) of the drug combinations was modeled with step-wise second-order linear regression analysis in Matlab^®^, generating regression coefficients describing the contribution of each drug (single drug first order; single drug second order, drug^2^) and drug-pair (drug-drug interaction) to the overall activity of the combinations.

### 4.4. Calculation of Clinically Used Doses (CUD)

The drug concentrations over time were acquired from pharmacokinetic studies performed in patients with the drugs given at standard or maximum tolerated doses. The concentration over the first 24 h, reported as the area under the curve (AUC_0–24 h_), was used to calculate the average drug concentration in that period. The CUDs were 0.49 µM for folinic acid [79], 9.61 µM for 5-fluorouracil [80], 0.39–0.59 µM for oxaliplatin [81,82] and 0.1 µM for irinotecan/SN-38 [81,83].

### 4.5. Statistical Analysis

All data are presented as the mean of multiple independent experiments with corresponding standard deviation (SD) as indicated in the figure legends. Data analysis was performed using Graphpad Prism^®^ version 8.0.1 using one-way or two-way ANOVA tests with post-hoc multiple comparison tests as specified in the figure legends. Statistical significance was indicated with * *p* < 0.05, ** *p* < 0.01 and *** *p* < 0.001.

## Figures and Tables

**Table 1:** CRC cell lines and drugs used in the study and their characterization [33,34,35,36,37].

**Cell Line**	**Duke’s Type**	**Cell Status**	**MSI/CIN**	**CIMP**	**Mutations/Deregulations**
DLD1	C	undifferentiated	MSI	CIMP ^+^	APC ^l1417fs, R2166^, KRAS ^G13D^, PIK3CA ^E545K; D549N^, TP53 ^S241F^
HCT116	A	undifferentiated	MSI	CIMP ^+^	KRAS ^G13D^, PIK3CA ^H1047R^
SW620	C	undifferentiated	MSS; CIN ^pos46^	CIMP ^−^	APC ^Q1338^, KRAS ^G12V^, TP53 ^R273H; P309S^
LS174T	B	colon-like	MSI	CIMP ^−^	KRAS ^G12D^, PIK3CA ^H1047R^, BRAF ^p.D211Gc^
**Drug**		**Chemical Formula**	**IUPAC Chemical Nomenclature**		**Abb.**
folinic acid (leucovorin)		C_20_H_23_N_7_O_7_	(2*S*)-2-[[4-[(2-amino-5-formyl-4-oxo-3,6,7,8-tetrahydropteridin-6-yl)methylamino]benzoyl]amino]pentanedioic acid		FA
5-fluorouracil		C_4_H_3_FN_2_O_2_	5-fluoro-1*H*-pyrimidine-2,4-dione		FU
oxaliplatin		C_8_H_12_N_2_O_4_Pt	[(1*R*,2*R*)-2-azanidylcyclohexyl]azanide;oxalate;platinum(4+)		OX
irinotecan		C_33_H_38_N_4_O_6_	[(19*S*)-10,19-diethyl-19-hydroxy-14,18-dioxo-17-oxa-3,13-diazapentacyclo[1 1.8.0.0_2,11_.0_4,9_.0_15,20_]henicosa-1(21),2,4(9),5,7,10,15(20)-heptaen-7-yl] 4-piperidin-1-ylpiperidine-1-carboxylate		IRI
7-Ethyl-10-hydroxycamptothecin *		C_22_H_20_N_2_O_5_	(19*S*)-10,19-diethyl-7,19-dihydroxy-17-oxa-3,13-diazapentacyclo[11.8.0.0_2,11_.0_4,9_.0_15,20_]henicosa-1(21),2,4(9),5,7,10,15(20)-heptaene-14,18-dione		SN

MSI: microsatellite instability; MSS: microsatellite stability; CIN: chromosomal instability, CIMP: CpG island methylator phenotype; Abb.: abbreviation; IUPAC: International Union of Pure and Applied Chemistry; * active metabolite of IRI, also known as SN38.

**Table 2:** Efficacy and combinatory index (CI) of FA/5-FU (FF) and FF/OX/SN in CRC cells.

Drug	DLD1	HCT116	SW620	LS174T
FA [µM] 5-FU [µM]	0.5 2.0	0.5 2.0	0.25 6.0	0.5 6.0
ATP levels [% CTRL] ±SD	83.2 2.1	83.4 3.8	91.5 4.5	86.6 4.9
CI _FF_	1.65	0.97	0.62	0.82

**Table 3:** Efficacy and combinatory index (CI) of four-drug mixtures at CUD and ODC doses.

Drug	CUD	DLD1 ODC	HCT116 ODC	SW620 ODC	LS174T ODC
FA [µM]	0.5	0.5	0.5	0.25	0.5
5-FU [µM]	10	2	2	6	6
SN [µM]	0.1	0.02	0.003	0.004	0.004
OX [µM]	0.6	0.5	0.2	0.3	0.2
ATP levels [% CTRL] ±SD	-	48 4.9	35 0.7	37 8.6	45 2.2
CI _FF/OX/SN_	-	1.61	2.60	3.45	2.47

CUD: clinically used dose (see Materials and Methods for dose conversion); ODC: optimized drug combination.

## Supplementary Materials

All research records and experimental data are available online. Figure S1. Isobolograms of (A) dose- and (B) median effect of FA/5-FU (FF) in all CRC cell lines, Figure S2. Isobolograms of drug doses (A) and median effect (B) for FF/OX/SN in CRC cells, Table S1. Combinatory index (CI) single drug doses and effect for FA/5-FU (FF) in CRC cells, Table S2. Combinatory index (CI) single drug doses and effect for FF/OX/SN in CRC cells.
