# Supplementary material for: Drug-Drug Interactions of Irinotecan, 5-Fluorouracil, Folinic Acid and Oxaliplatin and Its Activity in Colorectal Carcinoma Treatment
Source: Molecules. 2020 Jun 4;25(11):2614. doi: 10.3390/molecules25112614 (PMC7321123; doi:10.3390/molecules25112614)
Supplement: Supplementary file 1 [file molecules-25-02614-s001.pdf]

Supplementary information

Drug-drug interactions of irinotecan, 5-fluorouracil, folinic acid and oxaliplatin and its activity in colorectal carcinoma treatment

Marloes Zoetemelk[1,2#], George M. Ramzy[1,2#], Magdalena Rausch[1,2#], Patrycja Nowak-Sliwinska[1,2,*]

[1] Molecular Pharmacology Group, School of Pharmaceutical Sciences; Institute of Pharmaceutical Sciences of Western Switzerland, University of Geneva, 1 Rue Michel-Servet, 1211, Geneva 4, Switzerland

[2] Translational Research Center in Oncohaematology, 1 Rue Michel-Servet, 1211, Geneva 4, Switzerland

# Equal contribution

* Correspondence: Patrycja Nowak-Sliwinska, 1 Rue Michel-Servet, 1211 Geneva 4, Switzerland; Tel: +41 22 379 3352, e-mail: Patrycja.Nowak-Sliwinska@unige.ch

Supplementary Figure S1-S2
Supplementary Tables 1-2

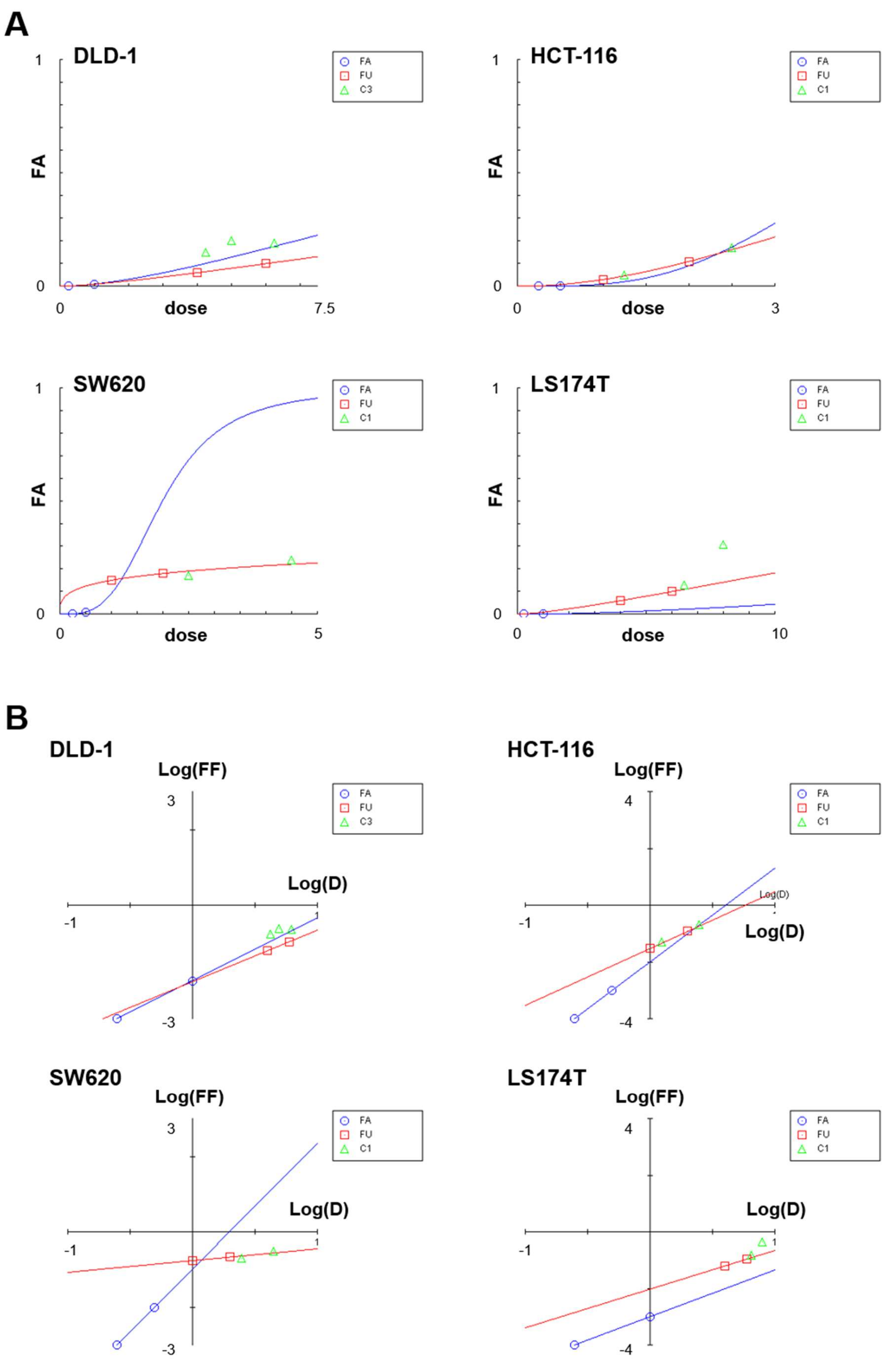

Supplementary Figure S1. Isobolograms of (**A**) dose- and (**B**) median effect of FA/5-FU (FF) in all CRC cell lines.

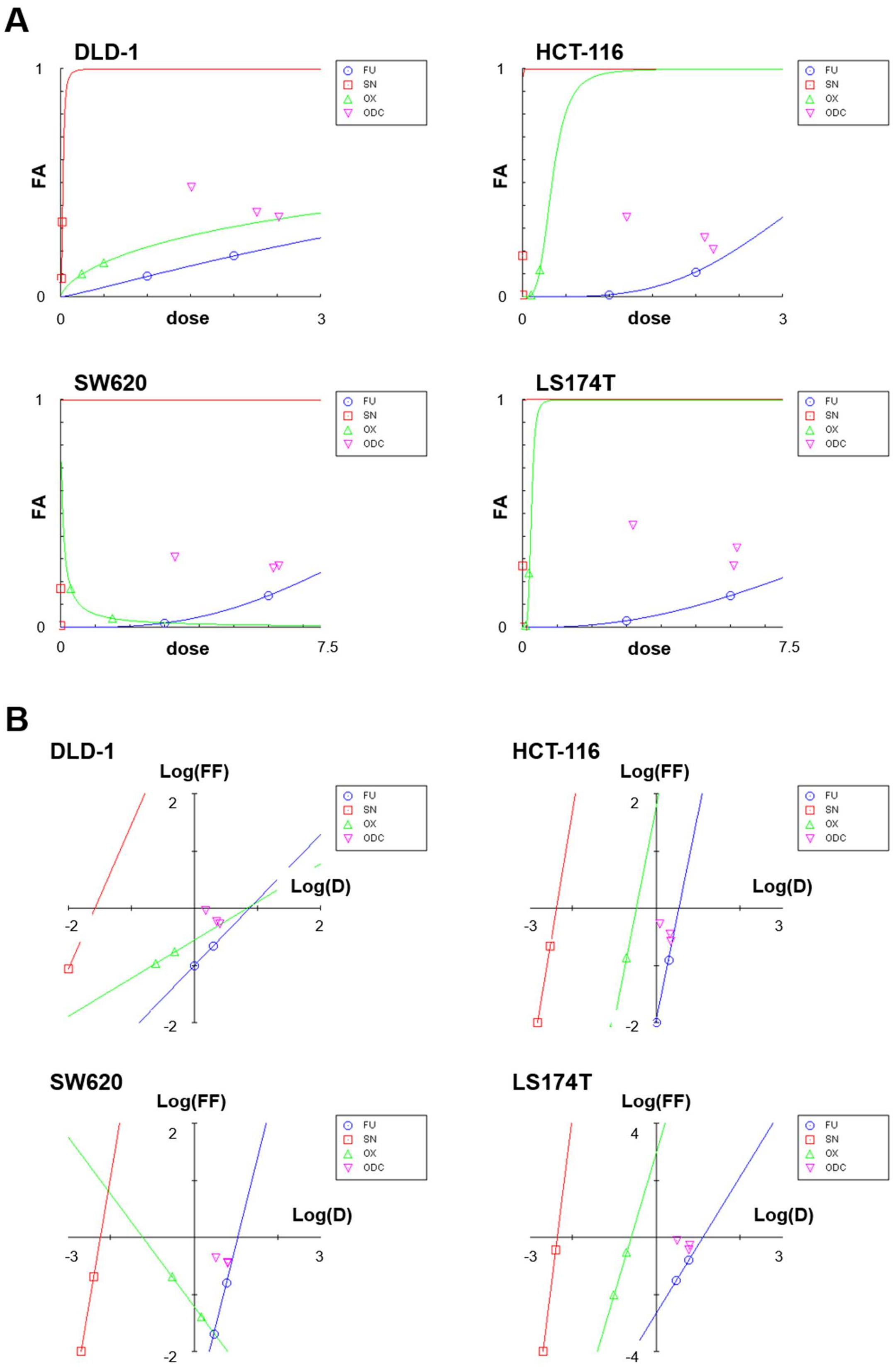

Supplementary Figure S2. Isobolograms of drug doses (**A**) and median effect (**B**) for FF/OX/SN in CRC cells.

Supplementary Table 1. Combinatory index (CI) single drug doses and effect for FA/5-FU (FF) in CRC cells.

**DLD1 CI $_{FF}$**		**HCT116 CI $_{FF}$**		**SW620 CI $_{FF}$**		**LS174T CI $_{FF}$**	
**Drug [µM]**	**effect**	**Drug [µM]]**	**effect**	**Drug [µM]**	**effect**	**Drug [µM]**	**effect**
FA 0.5	0.01	FA 0.5	0.001	FA 1.0	0.01	FA 1.0	0.001
FA 0.25	0.001	FA 0.25	0.0001	FA 0.25	0.001	FA 0.25	0.0001
FU 4.0	0.28	FU 2.0	0.11	FU 6.0	0.1	FU 6.0	0.1
FU 1.0	0.15	FU 1.0	0.03	FU 4.0	0.06	FU 4.0	0.06

Supplementary Table 2. Combinatory index (CI) single drug doses and effect for FF/OX/SN in CRC cells.

**DLD1 CI $_{FF/OX/SN}$**		**HCT116 CI $_{FF/OX/SN}$**		**SW620 CI $_{FF/OX/SN}$**		**LS174T CI $_{FF/OX/SN}$**	
**Drug [µM]**	**effect**	**Drug [µM]]**	**effect**	**Drug [µM]**	**effect**	**Drug [µM]**	**effect**
FA 0.5 + 5-FU 2.0	0.18	FA 0.5 + 5-FU 2.0	0.11	FA 0.25 + 5-FU 6.0	0.14	FA 0.5 + 5-FU 6.0	0.14
FA 0.25 + 5-FU 1.0	0.09	FA 0.25 + 5-FU 1.0	0.01	FA 0.13 + 5-FU 3.0	0.02	FA 0.25 + 5-FU 3.0	0.03
SN 0.02	0.33	SN 0.03	0.18	SN 0.004	0.17	SN 0.004	0.27
SN 0.01	0.08	SN 0.0015	0.01	SN 0.002	0.01	SN 0.002	0.001
OX 0.5	0.15	OX 0.2	0.12	OX 0.3	0.17	OX 0.2	0.24
OX 0.25	0.1	OX 0.1	0.01	OX 0.15	0.04	OX 0.1	0.01
